# Microbial Interactions in Food Fermentation: Interactions, Analysis Strategies, and Quality Enhancement

**DOI:** 10.3390/foods14142515

**Published:** 2025-07-17

**Authors:** Wenjing Liu, Yunxuan Tang, Jiayan Zhang, Juan Bai, Ying Zhu, Lin Zhu, Yansheng Zhao, Maria Daglia, Xiang Xiao, Yufeng He

**Affiliations:** 1 School of Food and Biological Engineering, Jiangsu University, Zhenjiang 212013, China; 18660310376@163.com (W.L.); tang1834178338@163.com (Y.T.); jiayanzhang1988@163.com (J.Z.); 1000005134@ujs.edu.cn (J.B.); ying307@126.com (Y.Z.); zhulin19820402@ujs.edu.cn (L.Z.); zhaoys@ujs.edu.cn (Y.Z.); xiaoxiang1@aliyun.com (X.X.); 2 International Research Center for Food Nutrition and Safety, Jiangsu University, Zhenjiang 212013, China; maria.daglia@unina.it; 3 Department of Pharmacy, University of Napoli Federico II, Via D. Montesano 49, 80131 Naples, Italy

**Keywords:** fermented foods, microbial interactions, synthetic microbial communities, core microorganisms, genome-scale metabolic network

## Abstract

Food fermentation is driven by microbial interactions. This article reviews the types of microbial interactions during food fermentation, the research strategies employed, and their impacts on the quality of fermented foods. Microbial interactions primarily include mutualism, commensalism, amensalism, and competition. Based on these interaction patterns, the safety, nutritional composition, and flavor quality of food can be effectively improved. Achieving precise control of fermented foods’ qualities via microbial interaction remains a critical challenge. Emerging technologies such as high-throughput sequencing, cell sorting, and metabolomics enable the systematic analysis of core microbial interaction mechanisms in complex systems. Using synthetic microbial communities and genome-scale metabolic network models, complicated microbial communities can be effectively simplified. In addition, regulatory targets of food quality can be precisely identified. These strategies lay a solid foundation for the precise improvement of fermented food quality and functionality.

## 1. Introduction

Fermentation is one of the oldest methods of food production. Under the action of microorganisms and enzymes, food ingredients undergo a series of biochemical reactions, thereby achieving a better texture, taste, and nutritional properties [1,2,3]. Microbial communities are dynamic but tend to stabilize over time, which is often driven by microbial interactions. These interactions collectively facilitate the synthesis of flavor compounds, the degradation of harmful substances, and the enhancement of nutritional content in fermented food [4,5,6].

Advancements in molecular biology and omics technologies have revealed that the quality and safety of fermented foods depend largely on the dynamic equilibrium of microbial communities and the complexity of microbial interactions [7]. Based on microbial interactions, the safety, nutritional composition, and flavor quality of fermented food can be effectively improved. For example, lactic acid bacteria (LAB) and yeasts can promote the transformation of flavor precursors, enhancing the aroma and taste of fermented foods; harmful microorganisms such as foodborne pathogens and molds, as well as harmful substances like nitrite, can be effectively inhibited; and the biosynthesis and accumulation of bioactive constituents, such as Vitamin B, γ-aminobutyric acid [8], and phenols, are also enhanced via interactions among LAB.

During the natural fermentation process, the microbial community is dynamic and complex [9,10]. Although the community as a whole is intricate, the most critical factor influencing the quality of fermented foods is the interaction among core microorganisms. Therefore, identifying and characterizing these core microorganisms is of paramount importance [11]. Traditional fermentation processes largely depend on the natural enrichment and succession of microorganisms in open environments [12]. However, the stability of microbial communities in fermented foods is influenced by various external factors, including raw material batches, environmental conditions (such as temperature, pH, and nutrient composition), and the hygiene status of processing equipment [13]. This instability has become one of the main obstacles to achieving precise intervention and standardized production in the current food fermentation industry [14]. For example, different batches of cereal raw materials such as barley and highland barley harbor distinct microbial community structures, which can lead to variations in fermentation performance and final product quality [15].

Emerging interdisciplinary strategies provide new pathways to overcome the aforementioned bottlenecks. Constructing synthetic microbial communities with core functional strains allows for the analysis of complex interaction networks and facilitates the transition from natural fermentation to targeted regulation [16]. For example, the synergistic interactions of the core Baijiu brewing community significantly enhanced the flavor quality of the liquor [17]. In addition, constructing genome-scale metabolic models (GSMMs) allows for the dynamic analysis of the interspecies metabolic division of labor and the optimization of product synthesis pathways [18,19]. These strategies not only enhance the quality and yield of the final product, but also enable the precise regulation of the fermentation process.

The existing reviews primarily discuss how microbial interactions in fermented foods affect food quality. The strategies for exploring microbial interaction mechanisms and facilitating the qualities of fermented foods have not been summarized yet. Therefore, the review systematically summarizes the main forms of microbial interactions in fermented foods. Based on omics technologies, synthetic communities, and genome-scale metabolic network models, the analytical strategies for complex microbial interaction mechanisms, as well as the quality improvement strategies of fermented foods, were generalized. Additionally, the effects of microbial interactions on the safety, nutrition, and flavor of fermented foods were further discussed.

## 2. Types of Microbial Interactions in Fermented Foods

**Figure 1:**
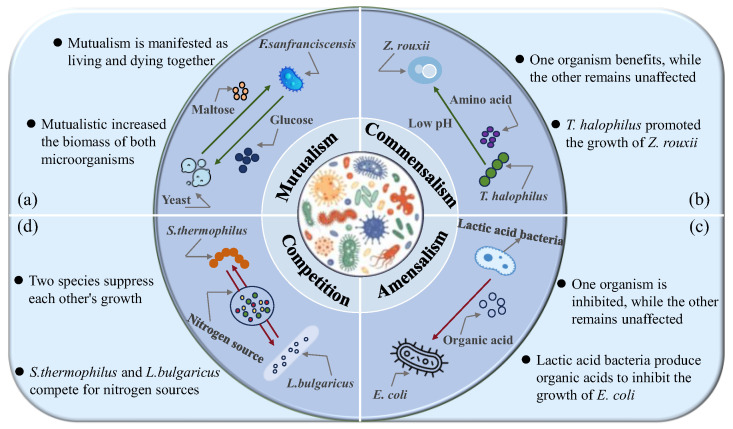
Forms of microbial interactions. (**a**) Mutualism. (**b**) Commensalism. (**c**) Amentalism. (**d**) Competition. “Green arrow” means that the growth of these microorganisms was promoted; “Red arrow” means that the growth of these microorganisms was inhibited. *F. sanfranciscensis*: *Fructilactobacillus sanfranciscensis*; *Z. rouxii*: *Zygosaccharomyces rouxii*; *T. halophilus*: *Tetragenococcus halophilus*; *E. coli*: *Escherichia coli*; *S. thermophilus*: *Streptococcu thermophilus*; and *L. bulgaricus*: *Lactobacillus delbrueckii* subsp. *bulgaricus*.

Food fermentation is the process through which microorganisms convert raw materials into energy and metabolic products [20,21]. Microbial communities in food fermentation systems primarily consist of four major types: bacteria, molds, yeasts, and actinomycetes [22,23]. These microorganisms create a complex network through dynamic interactions like synergistic symbiosis and metabolic competition. These complex communities can activate multiple biosynthetic pathways, including vitamin production, peptide transformation, and bioactive substance accumulation. Thereby, the nutrient compounds of fermented foods can be further improved [24]. The microbial interactions in fermented foods are primarily categorized into mutualism, commensalism, amensalism, and competition (Figure 1).

### 2.1. Mutualism

Mutualistic symbiosis refers to a close interaction between two or more microorganisms, where each party benefits from the relationship, demonstrating a phenomenon of living and dying together. Over 98% of microorganisms in nature are auxotrophic. Some key genes or pathways for synthesizing essential metabolites are missing in these microorganisms [25]. They must obtain the corresponding nutrients from their surrounding environment to sustain basic life activities. Therefore, mutualistic symbiotic relationships among microorganisms are widespread. In food fermentation systems, microorganisms can activate specific metabolic pathways, modulate gene expression, and enhance the production of metabolic end products through the exchange of metabolic intermediates or signaling molecules [26]. Among them, these products or signal molecules mainly include sugars, organic acids, amino acids, vitamins, quorum sensing signals, and iron carriers. For example, the Δ*trp2* strain of *Yarrowia lipolytica* and the Δ*trp4* strain of *Saccharomyces cerevisiae* form mutualism through the exchange of intermediates in tryptophan synthesis. The para-aminobenzoic acid secreted by the Δ*trp2* strain can be utilized by the Δ*trp4* strain, while the indole/tryptophan produced by the Δ*trp4* strain can supply the Δ*trp2* strain, mutually satisfying each other’s needs for tryptophan synthesis [27].

The mutual symbiosis between LAB and yeast is most common in fermented foods [28]. During grape juice fermentation, yeast provides LAB with amino acids like glutamine, while LAB supply yeast with usable carbon sources. The production of additional amino acids by yeast was further promoted. This symbiotic relationship significantly enhances the accumulation of flavor compounds [29]. In addition, a similar phenomenon was also observed during the sourdough fermentation process [30].

### 2.2. Commensalism

Commensalism refers to a relationship in which one organism’s growth is promoted, while the other organism’s growth is neither harmed nor benefited. Participants in commensalism exhibit less interdependence than those in mutualism. Commensal interactions generally occur in two main forms: (1) a metabolite produced by one organism during its growth is used by another organism, promoting its growth [31]; (2) an organism can consume a substance that inhibits another organism, thereby reducing the substance’s inhibitory effect on the latter and promoting its growth.

Commensalism can precisely regulate the quality of fermented foods by introducing one or more functional microorganisms without interfering with the growth and metabolic activities of the original fermentation strains. For example, during the fermentation process of blue cheese, *Penicillium molds* break down the proteins and fats in the cheese, producing substances such as amino acids, methyl ketones, and fatty acids [32]. These substances can promote the growth of LAB and the production of lactic acid. The acidification of cheese relies on the lactic acid produced by LAB. This commensal relationship enhances the taste, texture, and flavor of cheese, while also improving its preservation and safety [33]. During the soy sauce fermentation process, *Tetragenococcus halophilus* grows rapidly in the initial stage, metabolizing sugars to produce lactic acid, which lowers the system’s pH. This creates a growth advantage for the acid-tolerant *Zygosaccharomyces rouxii* [34]. Meanwhile, *T. halophilus* breaks down soybean protein to release free amino acids, providing a nitrogen source for *Z. rouxii*, thereby promoting its growth and the synthesis of flavor compounds (e.g., 4-ethylguaiacol) [35]. Since microorganisms are often densely clustered in communities (e.g., forming biofilms), competition for resources and space inevitably occurs among individuals, making it challenging to achieve truly unaffected growth.

### 2.3. Amensalism

Amensalism describes an interaction in which one organism’s growth is inhibited while the other remains unaffected, essentially forming an asymmetrical competition. The interaction takes place in two forms: (1) a metabolite produced by one organism during its growth, such as lactic acid or ethanol, inhibits the growth of another organism; (2) an organism can consume a substance that benefits another organism, thereby removing its positive effects and inhibiting the latter’s growth.

Amensalism is quite common in winemaking [36], kimchi fermentation [37], and dairy fermentation processes [38]. During kimchi fermentation, raw ingredients are often contaminated with *Escherichia coli*. LAB multiply significantly and produce acid [39,40]. The lactic acid generated exerts antibacterial effects, inhibiting the growth of *E.coli*, thus ensuring the normal progression of fermentation [41]. Cheng et al. [42] analyzed the amensalism between yeasts and LAB during the brewing process of Baijiu. They discovered that the ethanol, medium-chain fatty acids, proteins, and peptides secreted by *S. cerevisiae* and *Pichia anomala* significantly inhibited the growth of various LAB. This amensalism relationship enables yeast to gain a dominant position, thereby enhancing the production of desirable aroma compounds and reducing the formation of the unpleasant taste. In fermented dairy products, manganese ions can be absorbed by *Lacticaseibacillus rhamnosus* and *Lacticaseibacillus paracasei* subsp. *paracasei* from the fermentation substrate, which is required by spoilage organisms (e.g., *Cladosporium*). This amensalism effect further extends the shelf life of dairy products [43].

### 2.4. Competition

A competitive relationship involves two organisms mutually inhibiting each other’s growth [44]. Although certain microorganisms can inhibit the growth of other strains by secreting fermentation products, this inhibitory effect is typically transient. Among the inhibited microorganisms, resistant species often emerge, competing with dominant strains for nutrients and space, thereby diminishing their dominance.

Competition can be categorized into two main types based on differences in suppression mechanisms: exploitative competition and interference competition [45,46]. Exploitative competition is relatively passive and generally refers to species indirectly affecting each other’s growth by competing for limited shared resources, such as nutrients and space [45]. *Streptococcus thermophilus* and *Lactobacillus delbrueckii* subsp. *bulgaricus* are commonly regarded as having mutualistic interactions. However, competitive interaction between them has also been observed. Liu et al. [47] discovered that during yogurt fermentation, *S. thermophilus* grows rapidly in the early stage, requiring substantial nitrogen. As the pH decreases in the mid-stage, *L. bulgaricus* becomes more active due to its acid tolerance, and also increases its nitrogen demand. However, limited nitrogen availability leads to exploitative competition between the two species [48]. Interference competition is relatively proactive, referring to microorganisms that directly inhibit or harm competitors, such as by secreting antimicrobial substances, thereby disrupting their growth or survival [49]. Research has shown that during their growth, microorganisms actively secrete special metabolic products like antimicrobial peptides and bacteriocins to inhibit the growth of other strains. During the fermentation of sourdough, bacteriocins (e.g., paracasein SD1 [50] and bacteriocin BGSJ2-8 [51]) produced by *L. paracasei* subsp. *paracasei* can inhibit various foodborne pathogenic bacteria [52].

The complex interactions among microorganisms perform three major ecological functions in food fermentation: first, they optimize nutrient utilization and flavor compound synthesis through resource partitioning strategies; second, they establish cross-supply chains of metabolic products; and third, they create ecological niche barriers that prevent pathogen colonization. Strategies based on emerging technologies are needed to fully understand these interactions in complex food fermentation systems.

## 3. Strategy for Analyzing Microbial Interaction Mechanisms

**Figure 2:**
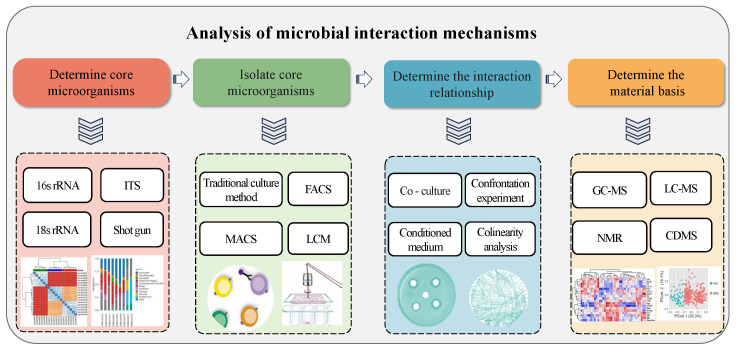
Analysis of the mechanisms of microbial interactions. ITS, internal transcribed spacer; FACS, fluorescence-activated cell sorting; MACS, immunomagnetic bead sorting; LCM, laser capture microdissection; GC-MS, gas chromatography–mass spectrometry; LC-MS, liquid chromatography–mass spectrometry; NMR, nuclear magnetic resonance; and CD-MS, charge detection–mass spectrometry.

The core microbiota dominates the fermentation process and significantly influences the nutrition and texture of fermented foods. Interactions among the core microbiota are a crucial driving force in maintaining community stability and functional diversity. Core species often show significant complementarity and interdependence in resource utilization, metabolite synthesis, and environmental adaptation. Based on these interactions, a dynamically balanced and functionally synergistic microbial community is formed. Therefore, exploring the interactions between core microbiota is essential for clarifying the distinct roles of different strains in substrate degradation, nutrient transformation, and flavor compound synthesis. Strategies for analyzing core microbial interaction are shown in Figure 2.

### 3.1. Identification of Core Microbiota

The core microbiota typically refer to those that remain consistently present during the fermentation process of a specific type of food [53]. For example, in fermented vegetables, the core microbiota primarily include LAB, *Leuconostoc*, *Lactococcus*, *Weissella*, and *Lactobacillus* [54]. For sausage fermentation, the main representative species are LAB, *Staphylococcus xylosus*, *Staphylococcus carnosus*, and *Debaryomyces hansenii* [55]. In fermented milk, the primary microorganisms are mainly *Lactococcus lactis*, *L.bulgaricus*, and *S. thermophilus* [56].

High-throughput sequencing (HTS) techniques are the primary methods for analyzing the core microbiota in food fermentation [57]. In the 1970s, the Sanger chain termination method was used to identify the bases present in DNA fragments and their correct sequence, known as first-generation sequencing technology [58]. However, a significant drawback of Sanger sequencing is its relatively low throughput and high cost [59]. Since then, the second-generation HTS technique has been developed. This technique utilizes large-scale parallel sequencing methods, significantly reducing the time and cost of processing large volumes of DNA samples [60]. Sequencing the human genome with this technology can be completed in two months at a cost of approximately USD 100,000 [61]. However, the second-generation HTS technique has a relatively short read length and relies on PCR amplification, leading to limited sequencing efficiency. In recent years, third-generation sequencing technology has emerged. This technology does not require PCR amplification and can read longer fragments [62].

Commonly used HTS methods for core microbiome identification include amplicon sequencing and metagenomic sequencing. Amplicon technology employs a polymerase chain reaction (PCR) to amplify specific conserved sequences of microorganisms. By analyzing the proportion of read counts, the relative abundance of microorganisms can be determined [35]. This method is applicable to nearly all sample types. Using fermented Pu’er tea as an example, Zhao et al. [63] employed amplicon sequencing technology (16srRNA sequencing, 18srRNA sequencing, and ITS region sequencing) to determine the bacterial and fungal composition in Pu’er tea. The results indicate that the core phylum in fermented Pu-erh tea is Proteobacteria, while the core fungi are primarily *Aspergillus*. Metagenomics conducts high-throughput sequencing on random fragments of all microbial DNA to obtain the comprehensive genetic information of microbial communities (e.g., species abundance and functional potential) [64]. Leech et al. [65] used shotgun metagenomics to analyze the microbial composition and functional potential of 58 fermented foods, including dairy products, water kefir, and sauerkraut. For example, the results for water kefir showed that the core genera include *Lactobacillus*, *Leuconostoc, Saccharomyces*, and *Acetobacter*. Genes related to vitamin synthesis, acid metabolism, antioxidation, carbohydrate degradation, and antibiotic resistance were also identified using this method. However, the above results are based on a single time point, making it difficult to identify core microorganisms Therefore, a more suitable strategy such as analyzing samples at different time points or the same analysis of similar samples is needed. Compared to amplicon sequencing, metagenomic sequencing exhibits a higher resolution of identification. In addition, metagenomic sequencing can overcome the restrictions of amplification primers.

However, a considerable proportion of taxa identified via HTS technology cannot be obtained through cultivation methods. The main reasons are as follows: (1) Cultivation methods can only obtain “cultivable live bacteria,” whereas sequencing captures the DNA of all microorganisms in the sample [66], including dead bacteria, lysed bacteria, and even free DNA. (2) The growth of certain microorganisms depends on specific symbiotic relationships or physicochemical conditions in their natural environment (e.g., low oxygen levels or host metabolites), which are challenging to fully replicate in laboratory culture conditions, resulting in some bacteria being unable to grow. (3) Amplicon sequencing is highly sensitive (capable of detecting low-abundance bacteria), but has limited resolution (usually at the genus level) [67], while cultivation methods are highly specific (at the species/strain level), but have low sensitivity (only detecting dominant bacteria).

### 3.2. Isolation of Core Microorganisms

Isolating representative strains of the core microbiota is a critical step in elucidating their interaction mechanisms. This isolation process is complex and systematic, primarily involving traditional cultivation methods and cell sorting techniques.

#### 3.2.1. Cultivation Methods

The traditional cultivation procedure includes enrichment culture, gradient dilution, strain screening, isolation, and preservation [68]. During this process, the medium’s components can be selectively controlled to promote the growth of the target microorganisms. For example, Kim et al. [69] successfully developed a novel selective medium (ABS), which supported the growth of 16 acetic acid bacteria while inhibiting the growth of 21 non-acetic acid bacteria. For the ABS medium, the key components for strain screening include acetic acid, ethanol, and penicillin. Using these components, acetic acid bacteria can grow selectively and form green colonies with yellow halos. Under traditional cultivation conditions, there are competitions among different microorganisms. This makes it difficult for non-dominant species to grow effectively during cultivation, and consequently, they cannot be isolated [70]. On the other hand, traditional cultivation techniques have limitations, such as long cultivation cycles and low sorting precision, resulting in low efficiency in microbial isolation.

#### 3.2.2. Cellular Sorting Techniques

In recent years, sorting techniques based on cellular characteristics have been increasingly employed for microbial screening. Fluorescence-activated cell sorting (FACS), laser capture microdissection (LCM), and immunomagnetic bead sorting (MACS) are widely used cell sorting techniques. FACS can screen cells that contain internal fluorescence or can be stained with fluorescent dyes. By setting specific wavelengths of excitation light to activate the fluorescent signals within the cells, target cells can be detected [71]. Gorter et al. [72] used two fluorescent dyes to stain different strains of *S.cerevisiae*, with each parent strain having a unique fluorescent marker. During hybridization, the fusion cells exhibit dual fluorescence. Subsequently, utilizing FACS, hybrid cells exhibiting dual fluorescent signals were identified and sorted based on their fluorescent signals, achieving the efficient separation of interspecies. However, FACS still has certain limitations; for example, the small size of bacteria necessitating highly sensitive detectors. In addition, cell damage may exist when the liquid stream is broken into individual droplets.

LCM enables the precise capture of target cells or tissue fragments by using low-energy infrared laser pulses to activate a thermoplastic film on the collection cap. In conjunction with specific labeling strategies such as fluorescence in situ hybridization probes and acid-fast staining, target cells can be recognized [73]. LCM can isolate single cells both in liquid suspensions [74] and solid samples (such as frozen sections of biofilms and paraffin-embedded tissues). Bracke et al. [75] used LCM to isolate contaminating microorganisms in milk. By adhering the target strain to the thermoplastic film and using low-energy infrared laser pulses, *Methylbacterium* species were identified as contaminant bacteria. However, due to the close contact between the cells in the sample and their neighbors, individual cells isolated via LCM are susceptible to contamination from the genetic material of surrounding cells [76].

MACS uses monoclonal antibodies attached to magnetic beads to bind specific antigens on the cell surface, thereby sorting the target strains [77,78]. Using superparamagnetic nanoparticles (typically with an iron oxide core coated with silica or polymer) with antibodies conjugated on the surface, specific bacteria can be targeted and captured. The advantages of MACS lie in its simple operation, low cost, and minimal impact on bacterial activity. Luciani et al. [79] developed immunomagnetic beads by conjugating a monoclonal antibody (MAb) that specifically recognizes the lipopolysaccharide of *E.coli* O104:H4. These immunomagnetic beads were added to the milk samples, where the MAb specifically binds to the target bacteria. Using these immunomagnetic beads, *E. coli* O104:H4 was effectively separated from the milk sample.

Cell sorting technology offers significant advantages over traditional cultivation techniques. This technology is more efficient, capable of rapidly processing large numbers of cells, and exhibits greater specificity [80]. Cell sorting technology overcomes the limitations of traditional cultivation methods that rely on cultivation conditions, enabling the separation of target cells from complex samples [81]. It is particularly suitable for studying microorganisms that are difficult to cultivate.

### 3.3. Determining Core Microbial Interactions

The interactions between two or more microorganisms can be explored through cultivation methods and symbiotic network analysis. Co-culture experiments, conditioned medium experiments, and confrontation experiments are reliable methods for intuitively determining these interaction relationships [82,83].

#### 3.3.1. Culture-Based Methods

Co-culture experiments are a classic method for characterizing microbial interactions. Researchers co-culture two or more microorganisms in vitro to observe changes in colony growth patterns, morphology, and metabolic products, thereby inferring the types of interactions between them. This provides a basis for accurately identifying the relationships between microorganisms. By comparing the monoculture and co-culture groups of *S. cerevisiae* and *Pichia stipitis*, the growth rate and cell density of *P. stipitis* in the co-culture group were higher than in the monoculture group, while no similar phenomenon was observed in *S. cerevisiae*. This indicates a commensal effect between the two species [84]. However, this method is inadequate for analyzing interactions involving multiple species. More complex weighting schemes are required to quantify the interactions.

Conditioned medium refers to a method of culturing one microorganism in the cell-free supernatant of another microorganism. Bacillus amyloliquefaciens [85] and Aspergillus oryzae [86] are commonly used in the fermentation of soybean paste [87], but their interactions within food matrices have not yet been explored. Digar et al. [88] investigated the interaction between these species using conditioned media and identified the amensalism effect. Specifically, when the fermentation metabolites of B. amyloliquefacien were extracted and *A. oryzae* was inoculated into the medium containing these metabolites, the hyphal growth and conidia formation of *A. oryzae* were inhibited. However, this inhibited effect was not observed in the cultivation of B. amyloliquefacien. Although oxidized lipids and surfactins have been identified as key metabolites in commensalism mediated by *A. oryzae* and *B. amyloliquefaciens*, the specific role of linear surfactins in Bacillus still requires further investigation.

The confrontation experiment involves inoculating the test bacteria and antagonistic bacteria separately on a solid medium to create a confrontation zone, thereby determining the antibacterial activity of the antagonistic bacteria. It is usually used for research on competitive relationships. Tian et al. [89] co-inoculated seven strains of *Trichoderma* and two strains of *Fusarium* on solid media to create 14 confrontation combinations. By observing the growth changes at the colony boundaries, they evaluated the antagonistic symbiotic interactions between them. The study found that *Trichoderma* significantly inhibited the growth of *Fusarium*, particularly forming a distinct inhibition zone in the contact area, and also reduced the production of mycotoxins.

#### 3.3.2. Co-Occurrence Network Analysis

The culture-based methods cannot simultaneously determine the complex interactions among multiple strains. They are usually used for determining the interactions between key species. Apart from culture-based methods, co-occurrence network analysis is also used for exploring interactions within microbial communities. This data analysis-based approach does not depend on cultivation experiments, making it ideal for examining interactions in complex microbial communities [90]. Co-occurrence networks identify co-occurrence patterns by calculating correlation coefficients, such as Pearson or Spearman coefficients. A positive correlation indicates that two species tend to occur under the same environmental conditions, possibly suggesting a synergistic relationship, while a negative correlation may imply competition or antagonistic relationships. The reliability of co-occurrence networks relies on data quality (such as sequencing depth and sample size) and the rationality of statistical methods [91]. Although co-occurrence networks can reveal the interactions between communities, static networks fail to capture the spatiotemporal dynamics of microbial communities. Furthermore, correlation analysis results are solely used to predict potential interactions, making it essential to further validate these interactions through cultivation experiments.

### 3.4. Determining the Material Basis for Microbial Interactions

Microorganism interaction involves a series of nutrient exchanges and metabolite productions. These substances can be categorized into three main types: carbon sources, nitrogen sources, and vitamins. The methods for determining these substances can mainly be divided into quantitative analysis and qualitative analysis [92].

#### 3.4.1. Quantitative Analysis

Currently, gas chromatography–mass spectrometry (GC-MS) and liquid chromatography–mass spectrometry (LC-MS) are key equipment for the identification and quantification of microbial interaction metabolites. GC-MS is a mass spectrometry technique primarily used for the separation and identification of volatile substances [93]. When analyzing non-volatile substances, a series of pre-treatment steps, such as derivatization, must be introduced before sample injection [94]. This makes the process relatively cumbersome and time-consuming, and it can easily introduce operational errors into the metabolomics data. Moreover, GC-MS is generally only suitable for detecting primary metabolites, and its range of detectable substances is not as extensive as that of LC-MS. Compared with GC-MS, LC-MS only needs simple sample preparation steps. In addition, it is suitable for the detection of macromolecular substances (such as proteins and polypeptides), non-volatile substances, and unstable substances [95]. Rong et al. [55] used LC-MS and GC-MS to monitor the changes in amino acids and branched-chain esters when *Lactiplantibacillus plantarum* was incubated in the culture supernatant of *D. Hansenii* and *S. xylosus*. They discovered that arginine, aspartic acid, cysteine, glutamine, glutamic acid, histidine, lysine, and proline were rapidly consumed after *L. plantarum* incubation, while an increase in the branched-chain ester content was observed.

#### 3.4.2. Qualitative Analysis

Qualitative analysis (also called untargeted metabolomics) can be used to identify the types of differential metabolites involved in the interaction process, thereby clarifying the key substances in microbial interactions [96]. In the same way as with quantitative analysis, CG-MS and LC-MS were also used for the qualitative detection of metabolites. Apart from mass spectrometers, qualitative analysis also relies on metabolite databases and multivariate statistical analysis [97,98]. Qualitative analysis usually focuses on small molecule metabolites. Zhang et al. [99] utilized untargeted metabolomics analysis to compare the metabolic differences between *S. cerevisiae* monoculture fermentation and co-culture fermentation with *L. plantarum* in dough, and a total of 604 metabolites in positive ion mode were identified, among which 139 significantly different metabolites were detected. Chan et al. [100] utilized LC-MS/MS-based non-targeted metabolome analysis to compare the differential metabolites during single-strain fermentation and the co-fermentation of coffee by *L. rhamnosus* and *S. cerevisiae*, and identified 37 differential metabolites, including 2-isopropylmalic acid, hydroxydodecanoic acid, and 2-isopropylmalate.

Metabolomics is often combined with other omics techniques. By integrating genomics to determine the possible metabolic pathways of microorganisms, transcriptomics to analyze gene expression, proteomics to identify key enzymes involved in metabolism, and then using metabolomics to detect the actual metabolites produced, the material basis of microbial interactions can be deeply analyzed from multiple levels. Bechtner et al. [101] studied the interaction mechanism between *Liquorilactobacillus nagelii* and *S. cerevisiae* using metagenomics combined with proteomics in water kefir. Genomics identified that *L. nagelii* possesses complete pathways for amino acid and riboflavin biosynthesis. Proteomic analysis revealed that, in co-culture with *S. cerevisiae*, *L. nagelii* showed 73 differentially expressed proteins—including the downregulation of riboflavin synthase and glutamate synthase and upregulation of amino acid transporters—indicating the uptake of yeast-derived nutrients.

## 4. Quality Regulation Strategies for Fermented Foods Based on Microbial Interactions

Interactions among core microorganisms mediate the formation of food qualities. Targeting these interactions is a feasible approach to regulating the quality of fermented foods [102]. The stable simulation of microbial communities in fermented foods and the precise identification of regulatory targets can be achieved using synthetic microbial communities and genome-scale metabolic network models [103]. The quality regulation strategies can be summarized as follows: whole-genome sequencing and the annotation of core microorganisms; constructing GSMMs and identifying regulatory targets; and using the synthetic microbial community to verify the effectiveness of regulatory targets.

### 4.1. Acquiring and Analyzing Microbial Genome

The microbial genome contains its entire metabolic potential. Using HTS technology, the complete information of the microbial genome can be obtained [57,104]. To fully harness the potential of genome sequences, it is essential to annotate them with biologically relevant information. Genome annotation primarily encompasses the annotation of nucleic acids, proteins, and biological metabolites [105]. Nucleic acid annotation primarily focuses on identifying the locations and structures of coding genes, non-coding genes, and their respective regulatory regions [106]. Protein annotation mainly involves studying the functions of coding genes [107]. Metabolic annotation is responsible for explaining the pathways through which genes and proteins are involved in metabolism [108].

Genomic annotation is a highly complex and data-intensive task that relies on a variety of efficient and accurate bioinformatics tools and platforms [109]. The Kyoto Encyclopedia of Genes and Genomes (KEGG) [110], Gene Ontology (GO) [111], and Clusters of Orthologous Groups of proteins (COG) [112] are three commonly used annotation tools. KEGG can be used to analyze gene biological information at the system level, including metabolic pathways, genetic information, and complex biological processes. GO is a comprehensive database describing gene functions, consisting of three parts: the biological process, cellular component, and molecular function. COG is constructed based on species protein sequences and is used for protein function annotation, classification, and evolutionary analysis.

High-quality genomic data can not only predict the individual metabolic capabilities of strains, but also reveal potential metabolic dependencies, cross-feeding, and resource competition among different strains [113]. Tang et al. [114] screened 2500 bacterial genomes for L-lysine production based on the presence of *lysA* gene, with *dapF* gene or *ddh* gene as supplementary markers. They identified 31 candidate strains, and 30% showed increased L-lysine levels in experiments. The fermentation of chickpeas with the top two strains (*B. amyloliquefaciens* and *L. paracasei* subsp. *paracasei*) led to a 43% increase in the L-lysine content in chickpea milk. For example, the presence or absence of B vitamin synthesis gene clusters (e.g., nicotinamide and niacin) in yeast may determine its supportive role for LAB in co-culture systems. Conversely, genes related to organic acid synthesis and amino acid transport in the LAB genome affect its potential to supply nutrients to yeast [29]. In addition, genome annotation can offer a foundation for the rational design of synthetic communities.

### 4.2. Establishing Genomic Metabolic Model

**Figure 3:**
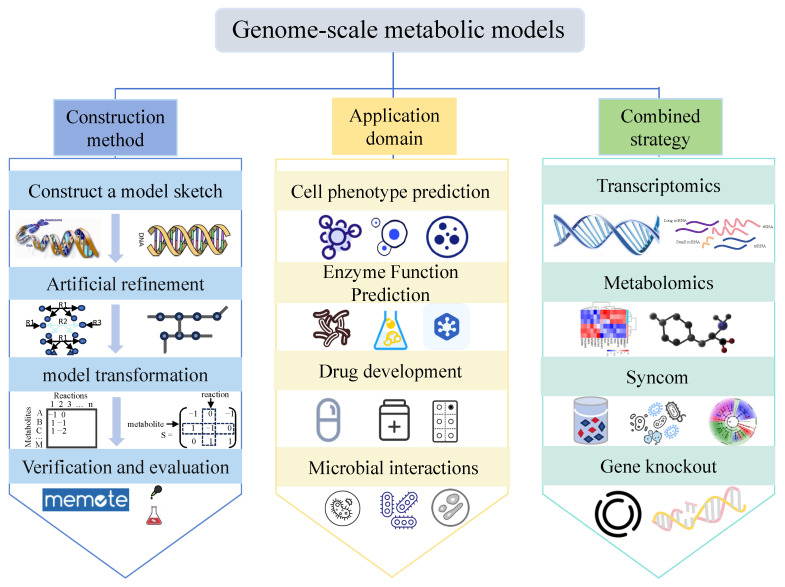
The construction methods and applications of GSMM.

The genome-scale metabolic model (GSMM) is a dynamic metabolic model that offers a comprehensive and precise description of cellular metabolic processes. It is founded on the complex interactions among a microorganism’s genes, proteins, and reactions, integrating stoichiometry and energy balance principles [115]. The construction and application of the GSMM are shown in Figure 3. A four-step tutorial for constructing the GSMM was recommended, which includes creating a model draft (coarse model), refining the model, converting it into a mathematical form, and evaluating the network [116]. The GSMM not only clearly reveals the relationship between genotypes and phenotypes, but also predicts changes in cellular metabolic flux under varying conditions [117]. Studies have demonstrated that integrating the GSMM and flux balance analysis (FBA) allows for the optimization of the medium to create the most suitable conditions for microbial growth. Based on the GSMM and FBA, Fan et al. [118] developed a minimal medium that can maintain cell growth. By removing components such as L-glycine, L-cysteine, L-methionine, thiamine, and pantothenic acid from a fully chemically defined medium, the minimal medium achieved 96.5% of the target product yield.

Genome sequencing data can be used to construct the GSMM of core microorganisms. Based on a single model, the flux balance analysis algorithm can be employed to integrate and simulate the metabolite flux distribution of different strains under co-culture conditions. This approach can predict the resource competition and metabolic complementarity relationships among the strains in such settings [119]. The GSMM can be used to screen key metabolic pathways for target metabolites. By targeting key metabolic pathways, the production of target products can be optimized and regulated through genetic modification, metabolite supplementation, and other methods [120]. Studies have shown that *Aureobasidium pullulans* can produce polymalic acid (PMA), and that PMA hydrolyzes to release the monomer L-malic acid (MA) [121]. Feng et al. [122] developed a cost-effective, eco-friendly process for PMA and MA production from sugarcane molasses. Using the GSMM of *A. pullulans*, the researchers predicted the reductive TCA cycle as the optimal pathway for PMA synthesis. When glucose or sucrose was used as the carbon source, the PMA synthesis rate was 15 mmol/gDW/h via the reductive TCA cycle, compared to 12.89 mmol/gDW/h through the oxidative branch. The model also identified pyruvate carboxylase (encoded by the *pyc* gene) as a key enzyme in the reductive TCA cycle. Based on this prediction, the authors constructed a *pyc* overexpression strain (FJ-PYC) and performed batch fermentation using sugarcane juice. The FJ-PYC strain achieved a PMA yield of 31.5 g/L and an MA yield of 36.5 g/L, representing a 15.1% increase compared to in the wild type. Schwalm et al. [123] used the GSMM to predict the hydrogen production of 298,378 microbial co-culture combinations. The combination of *Clostridium beijerinckii* and *Yokenella regensburgei* achieved the highest hydrogen production. According to the model, the increase in hydrogen production is attributed to *Y. regensburgei* promoting the growth of *C. beijerinckii* by producing lactic acid. As the cultivation experiments showed, the optical density and hydrogen production of *C. beijerinckii* increased as the lactic acid in the medium was depleted. These results further verified the predictions of the GSMM.

### 4.3. Simplifying Complex Microbial Communities

Microbial communities in traditional fermented foods are often complex and easily affected by raw material batches and environmental factors (such as temperature, pH, and nutrient composition) [124]. In complex microbial communities, it is typically a few core microorganisms that determine food quality. Therefore, core microorganisms can be used to simplify the microbial community and enhance its stability. Constructing a synthetic community is a key strategy for simplifying the complex community and realizing the regulation of fermentation products [125].

Synthetic microbial communities are microbial communities formed by assembling multiple microorganisms with well-defined genetic backgrounds under specific conditions. These communities offer advantages such as low complexity, high controllability, and strong stability [126,127]. The ultimate goal of synthetic microbial communities is to harness the individual capabilities of single microorganisms and their interactions to distribute labor for complex metabolic tasks, thereby achieving goals such as enhanced population productivity, stability, and metabolic functionality [128]. Five core bacterial species were identified based on the symbiotic characteristics, relative abundance, and flavor-producing capabilities of microorganisms in traditional fermented sausages. These strains were used to construct a synthetic community of sausage fermentation. As a result, sausages fermented via this synthetic community produced more floral, fruity, sweet, and fresh aromas compared to those produced via commercial fermentation tanks [129].

Based on the identification of key metabolic pathways and the corresponding products of microorganisms during fermentation using the GSMM, researchers can strategically design the species composition and metabolic roles of synthetic communities. The biosynthesis of specific functional metabolites can be selectively enhanced by targeting and regulating the material–energy transfer network between core microorganisms. Goncalvese et al. [130] analyzed 270 metagenome-assembled genomes (MAGs) from the Campos rupestres ecosystem in Brazil and designed synthetic microbial communities (SynComs) using a multi-genome metabolic modeling approach. Through targeted screening, they constructed a minimal community (MinCom) that was approximately 4.5 times smaller than the original while retaining key plant growth-promoting traits (PGPTs), such as iron acquisition, exopolysaccharide production, potassium solubilization, and nitrogen fixation. The final core species, mainly from four phyla (including Cyanobacteria, Eremiobacterota, Proteobacteria, and Verrucomicrobiota), provide a theoretical foundation and potential application for improving crop yield and stress resistance. While the study demonstrates the potential of metabolic modeling in SynCom design, its application in fermented food systems remains limited and warrants further investigation.

## 5. Impacts of Microbial Interactions on Fermented Foods

Microbial interactions exert significant effects on the safety, nutritional properties, and sensory quality of fermented foods. Some related studies are listed in Table 1**.**

### 5.1. Enhancing the Safety of Fermented Foods

In traditional and natural fermentation processes, the open environment poses potential safety risks [142]. It is estimated that hundreds of diseases are related to the consumption of contaminated food, which causes a global health burden [143]. The amensalism between different species can effectively inhibit the growth of pathogenic microorganisms, including pathogenic bacteria and pathogenic fungi, thereby enhancing the safety of fermented foods.

#### 5.1.1. Pathogenic Bacteria

During the food fermentation process, LAB produce metabolites such as organic acids [144], hydrogen peroxide [145], and bacteriocins [146,147]. These metabolites exhibit a significant inhibiting effect on foodborne pathogenic bacteria, such as *Bacillus cereus* [148], *S. typhimurium* [149], *L. monocytogenes* [150,151], and *S. aureus* [152].

LAB can produce various organic acids, which lower the environmental pH and inhibit the growth of pathogenic bacteria, exhibiting non-specific antibacterial effects [153]. For example, *L. lactis* can inhibit the growth of *Salmonella* and *E. coli* by producing formic acid [154]. Bacteriocins are antimicrobial peptides produced by various types of bacteria [147]. Their antibacterial mechanisms mainly fall into two categories: (1) Class I bacteriocins primarily function by inhibiting the synthesis of peptidoglycan, and (2) Class II bacteriocins work by forming pores that disrupt the stability of the cytoplasmic membrane [155]. Additionally, some bacteriocins function as hemolysins [156], breaking down bacterial cell walls that are typically composed of peptidoglycan, resulting in cell lysis. Chang et al. [131] used *Limosilactobacillus citreum* G17 to prepare kimchi, and the bacteriocin it produced could directly inhibit the growth of foodborne pathogens such as *E. coli* O157:H7 and *S.aureus* by disrupting the pathogenic bacterial cell membrane and inhibiting cell wall synthesis. LAB can also produce many other small molecules, such as hydrogen peroxide. Its antibacterial mode of action is achieved by inactivating key enzymes, resulting in a change in their catalytic activity [157]. Delbes-Paus et al. [132] added the cell-free supernatant of *Lactococcus garvieae* to raw bovine milk and found that it inhibited the growth of *S. aureus*. However, after the addition of catalase, the antibacterial effect was weakened. These results indicate that *L. garvieae* inhibits the growth of *S. aureus* by producing hydrogen peroxide.

#### 5.1.2. Pathogenic Fungi

Fungi are microorganisms widely found in nature. Compared to bacterial contamination, pathogenic fungi are commonly present in grains [158], nuts [159], and fruits [160]. The main pathogenic mode of fungi is the production of mycotoxins (e.g., aflatoxins [161] and ochratoxins [162]), posing a serious threat to food safety. LAB and yeast significantly inhibit fungi during food fermentation.

On the one hand, the organic acids produced by LAB during fermentation can directly inhibit the germination of mold spores and the growth of mycelium [163]. Cizeikiene et al. [164] isolated five strains of LAB from rye sourdough. These strains can produce acetic acid, lactic acid, and bacteriocin-like inhibitory substances (BLISs), which exhibit inhibitory effects against various fungi and yeasts. Single-cell suspensions of these strains were sprayed onto the surface of bread, effectively inhibiting fungal growth for up to 8 days of storage. On the other hand, volatile organic compounds (VOCs) such as alcohols, aldehydes, and esters, which are produced by yeast during fermentation, have been shown to effectively inhibit the growth of pathogenic fungi and the synthesis of their toxins [165]. Certain yeast strains can indirectly inhibit mold proliferation by competing for nutrients or colonization sites, thus creating resource competition. Öztekin et al. [133] evaluated the control efficacy of three strains—*Hanseniaspora uvarum*, *M. guilliermondii*, and *Metschnikowia* aff. *Pulcherrima*—against *Penicillium* on citrus fruits by elucidating their mechanisms of action. They found that *M. guilliermondii* exhibited the highest biofilm formation (OD_600_ of 0.93 ± 0.01) and antifungal activity (71.13%) through the production of VOCs.

### 5.2. Enhancing the Nutritional Value of Fermented Foods

Fermented foods usually have a unique nutritional composition, with the metabolic activities of microorganisms being the key factor. In co-fermentation or natural complex microbial communities, the nutritional feedback and metabolic network coupling among different microorganisms can significantly enhance the enrichment of nutrients (e.g., dietary fiber and vitamins) and produce bioactive components (e.g., phenolic compounds), ultimately enhancing the nutritional value of traditional fermented foods [166,167].

#### 5.2.1. Dietary Fiber

Dietary fiber refers to the carbohydrate in food that cannot be absorbed or digested by the body’s endogenous enzymes [168]. Consuming an appropriate amount of dietary fiber can regulate gut dysbiosis, reduce cholesterol levels, and lower the risk of cardiovascular diseases [169,170]. Dietary fiber is classified into soluble and insoluble types based on their solubility [168,171]. Compared with insoluble types, soluble dietary fiber is more easily utilized by gut microbiota, thereby exerting the potential prebiotic effects.

Microbial interactions can significantly enhance the ability to deconstruct and reutilize the structures of dietary fibers in raw materials. For example, *Y. lipolytica* [172] is an obligate aerobic yeast that can secrete proteases, peptidases, and lipases. *R. microspores* [173] is an aerobic food-grade fungus that produces a variety of extracellular enzymes, including carbohydrate enzymes, proteases, lipases, and phosphatases. Vong et al. [134] employed the co-fermentation of okara with *Y. lipolytica* and *R. microsporus*. Compared to the unfermented okara, the co-fermented group exhibited a 33% reduction in insoluble dietary fiber and a 176% increase in soluble dietary fiber content. These results suggest a substrate-complementary synergistic effect between the two microorganisms, significantly enhancing the nutritional value of okara.

#### 5.2.2. Phenolic Compounds

Phenolic compounds consist of one or more aromatic rings containing hydroxyl groups and are typically classified into flavonoids, phenolic acids, stilbenes, polyphenol amides, coumarins, and tannins [174]. Phenolic compounds exhibit physiological activities such as free radical scavenging and antibacterial, anti-inflammatory, and anti-aging properties [175,176]. However, phenolic compounds are mostly found in their natural state as esterified, glycosylated, or polymerized forms, which results in low bioavailability. Microbial interactions during the fermentation process provide a crucial pathway for the release, transformation, and enhancement of the activity of phenolic compounds. Wang et al. [135] used *Monascus purpureus* GIM 3.592 and *S. cerevisiae* GIM 2.139 for co-fermentation. The content of phenolic compounds was found to be increased by 2.06 times compared to unfermented samples. This result was achieved through the synergistic metabolism and enzymatic action between the two microorganisms. Research by Puspitasari et al. [177] demonstrates that the synergistic solid-state fermentation of Moringa seeds with *A. oryzae* and *A. niger* significantly increased the bioactive component content of the fermentation products. Compared to single-strain fermentation, this synergistic approach enhances the availability of total phenolics by nearly 50%, significantly boosting the antioxidant activity of Moringa seeds. We speculate that this increased phenolic availability may result from the conversion of bound phenolics—such as those linked to dietary fiber—into their free forms.

#### 5.2.3. Vitamins

Vitamins are micronutrients that are essential for various metabolic processes and the normal functioning of the human body [178,179]. Only a few vitamins can be synthesized within the human body. Therefore, increasing the vitamin content of fermented foods has dietary significance. Research has found that LAB can activate vitamin synthesis pathways through mutualism. Lactic acid is the main metabolite of *Bifidobacterium animalis* subsp. *lactis* and also the preferred carbon source for *Propionibacterium*. Meanwhile, *Propionibacterium* can produce growth stimulants, such as 1,4-dihydroxy-2-naphthoic acid (DHNA), which can enhance the growth of *Bifidobacterium animalis* subsp. *lactis* [136,137]. Their co-culture can increase the content of vitamin B12 [138], which is a micronutrient essential for DNA synthesis [180,181]. The co-cultivation of LAB and propionic acid bacteria has yielded excellent results in folic acid production. Experimental results indicate that the total folic acid yield can reach 8400 ng/mL under co-cultivation, which is comparable to the capabilities of genetically engineered strains [182].

### 5.3. Improving the Flavor Compounds of Fermented Foods

During fermentation, the flavor of fermented foods depends on complex intra- and inter-species interactions. Single species with limited metabolic gene functions often encounter challenges such as intracellular resource imbalance and the uneven distribution of metabolic flux. This defect further hinders the production of flavor compounds. Constructing synthetic communities with metabolic interactions has been regarded as a promising approach for the production of flavor compounds like organic acids, esters, and amino acids [183].

#### 5.3.1. Organic Acid

Organic acids are important flavor compounds in traditional fermented foods, characterized by strong aromas such as fresh, fruity, and nutty notes [184]. Organic acids can be divided into two categories: volatile acids and non-volatile acids. Volatile acids include formic acid, acetic acid, propionic acid, butyric acid, 3-methylbutyric acid, valeric acid, and caproic acid, which are characterized by strong pungency and a short aftertaste. Non-volatile acids include lactic acid, tartaric acid, citric acid, malic acid, succinic acid, and fumaric acid, which can regulate acidity and impart a mild taste to fermented foods [185]. Studies have shown that metabolic complementation exists between yeast and other microorganisms, which increases the content of organic acids. Wen et al. [139] used *D. Hansenii* in co-fermentation with *L. plantarum*, *Latilactobacillus sakei*, and *Latilactobacillus curvatus* for sausage fermentation. They found that the levels of tartaric acid, lactic acid, and citric acid in the co-fermentation groups nearly doubled. *D.Hansenii* releases amino acids through protease activity, which can be utilized by LAB, while the organic acids metabolized by LAB can be utilized by yeast. The co-fermentation creates a metabolic complementarity between species.

#### 5.3.2. Esters

Esters possess typical fruity characteristics and are key substances influencing the flavor of fermented foods. Branched-chain esters, particularly ethyl 2-methylpropanoate, ethyl 3-methylbutanoate, and ethyl 2-methylbutanoate, play a crucial role in imparting fruity and floral aromas to the final product [186]. Research indicates that yeast can produce extracellular proteases to release amino acids, thereby promoting bacterial growth and increasing the production of branched-chain esters. In fermented sausages, coagulase-negative staphylococci, LAB, and yeast are predominant and play a major role in ester production [187]. Rong et al. [140] used *D. Hansenii* and *S. xylosus* for both the single and co-fermentation of sausages. They discovered that *D. Hansenii* provided arginine, aspartic acid, cysteine, glutamine, glutamic acid, histidine, lysine, and proline, which served as the main drivers for the growth of *S. xylosus*. This, in turn, promoted the accumulation of branched-chain esters in *S. xylosus*, tripling the content of these esters.

#### 5.3.3. Amino Acid

Amino acids are important umami flavor components in fermented foods. For microbial metabolism, amino acids exert various physiological functions, including energy supply, nitrogen balance regulation, biosynthesis, and neurotransmitter synthesis [188]. Enhanced protein hydrolysis and substrate sharing may occur in food fermentation, leading to the accumulation of amino acids. LIU S et al. [141] used *R. oryzae*, *A. niger*, *Mucor*, and *S. cerevisiae* to perform mixed fermentation of rice wine. The levels of amino acid nitrogen, umami, and sweet free amino acids were all higher than those in rice wine fermented with wheat koji, leading to better sensory performance.

## 6. Conclusions and Future Prospects

Microbial interactions reshape the community structure in fermented foods through mutualism, commensalism, amensalism, and competition, thus affecting the safety, nutritional value, and sensory quality of products. Using omics technologies, the mechanisms of microbial interactions can be elucidated. The precise regulation of fermentation may be achieved through metabolic network models and synthetic communities. However, some shortcomings of the current research still need to be further addressed. For example, species-level sequencing of the microbial community is needed in further study. In addition, the identification of core microbiota should rely on the dynamic results of the microbiota, rather than the end-point sequencing of microbiota. In the future, food fermentation should be understood from an ecological perspective, with increased focus on culturomics to explore unknown species, longitudinal studies of microbial dynamic interaction networks, and processing data with advanced computational tools. These efforts will provide a feasible solution for the precise improvement of fermented food quality and functionality.

## Figures and Tables

**Table 1:** Impacts of microbial interactions on fermented foods.

Advantages	Products	Microorganisms	Form	Effects	References
Safety enhancement	Kimchi	*Limosilactobacillus citreum* *Escherichia coli* *Staphylococcus aureus*	Competition	Bacteriocin produced by *L. citreum* inhibits *E. coli* and *S. aureus*.	[131]
	Raw bovine milk	*Lactococcus garvieae* *Staphylococcus aureus*	Amensalism	*L. garvieae* inhibits the growth of *S. aureus* by producing hydrogen peroxide.	[132]
	Citrus	*Meyerozyma guilliermondii* *Penicillium digitatum*	Amensalism	Volatile organic compounds produced by *M. guilliermondii* can inhibit 71.13% of *P. digitatum*.	[133]
Nutritional quality enhancement	Okara	*Yarrowia lipolytica* *Rhizopus oligosporus*	Mutualism	Co-fermentation resulted in a 176% increase in soluble dietary fiber content.	[134]
	Moringa seeds	*Monascus purpureus* *Saccharomyces cerevisiae*	Mutualism	Co-fermentation resulted in a 2.06-fold increase in phenolic content.	[135]
	Soy whey	*Bifidobacterium animalis* subsp. *lactis* *Propionibacterium freudenreichii*	Mutualism	Co-fermentation of two strains enhanced vitamin B12 production.	[136,137,138]
Sensory enhancement	Sausage	*Debaryomyces hansenii* *Lactobacillus* spp.	Mutualism	Co-fermentation increased the contents of tartaric acid, lactic acid, and citric acid.	[139]
	Sausage	*Debaryomyces hansenii* *Staphylococcus xylosus*	Commensalism	Co-fermentation of two strains increased the content of branched-chain esters.	[140]
	Rice wine	*Saccharomyces cerevisiae* *Aspergillus niger* *Mucor* spp. *Rhizopus chinensis*	Mutualism	Co-fermentation increased the contents of amino acid nitrogen, umami, and free amino acids, achieving a better sweet taste.	[141]

## Data Availability

No data was used for the research described in the article.
